# Immunomodulatory Effects of Azithromycin Revisited: Potential Applications to COVID-19

**DOI:** 10.3389/fimmu.2021.574425

**Published:** 2021-02-12

**Authors:** Vincent J. Venditto, Dalia Haydar, Ahmed Abdel-Latif, John C. Gensel, Michael I. Anstead, Michelle G. Pitts, Jarrod Creameans, Timothy J. Kopper, Chi Peng, David J. Feola

**Affiliations:** ^1^Department of Pharmaceutical Sciences, College of Pharmacy, University of Kentucky, Lexington, KY, United States; ^2^Department of Bone Marrow Transplantation and Cellular Therapy, St. Jude Children's Research Hospital, Memphis, TN, United States; ^3^Gill Heart Institute and Division of Cardiovascular Medicine, College of Medicine, University of Kentucky, Lexington, KY, United States; ^4^Department of Physiology, Spinal Cord and Brain Injury Research Center, College of Medicine, University of Kentucky, Lexington, KY, United States; ^5^Department of Pediatrics, College of Medicine, University of Kentucky, Lexington, KY, United States; ^6^Department of Pharmacy Practice and Science, College of Pharmacy, University of Kentucky, Lexington, KY, United States

**Keywords:** immunomodulation, COVID-19, azithromycin, inflammation, therapeutic

## Abstract

The rapid advancement of the COVID-19 pandemic has prompted an accelerated pursuit to identify effective therapeutics. Stages of the disease course have been defined by viral burden, lung pathology, and progression through phases of the immune response. Immunological factors including inflammatory cell infiltration and cytokine storm have been associated with severe disease and death. Many immunomodulatory therapies for COVID-19 are currently being investigated, and preliminary results support the premise of targeting the immune response. However, because suppressing immune mechanisms could also impact the clearance of the virus in the early stages of infection, therapeutic success is likely to depend on timing with respect to the disease course. Azithromycin is an immunomodulatory drug that has been shown to have antiviral effects and potential benefit in patients with COVID-19. Multiple immunomodulatory effects have been defined for azithromycin which could provide efficacy during the late stages of the disease, including inhibition of pro-inflammatory cytokine production, inhibition of neutrophil influx, induction of regulatory functions of macrophages, and alterations in autophagy. Here we review the published evidence of these mechanisms along with the current clinical use of azithromycin as an immunomodulatory therapeutic. We then discuss the potential impact of azithromycin on the immune response to COVID-19, as well as caution against immunosuppressive and off-target effects including cardiotoxicity in these patients. While azithromycin has the potential to contribute efficacy, its impact on the COVID-19 immune response requires additional characterization so as to better define its role in individualized therapy.

## Introduction

Azithromycin is administered to over 40 million patients annually for its antibacterial activity (1), but characterization of the immunomodulatory properties of the macrolide antimicrobials has expanded their use. Clinically, azithromycin is used to treat bacterial infections of the upper respiratory tract, but has also been shown to improve lung function in subjects with various pulmonary pathologies, most notably in patients with cystic fibrosis (2–6). Mechanistic studies demonstrate immunomodulatory activity through the regulation of cellular processes involved in inflammation including NF-κB signaling (7–12), inflammasome activation (13, 14), and autophagy flux (15, 16). Although azithromycin inhibits a variety of pro-inflammatory pathways, it does not result in full immune suppression as is induced by glucocorticoids and other immunosuppressive therapies. Rather, azithromycin exhibits immunomodulatory properties by shifting the inflammatory response, mainly in macrophages, toward one characterized by functional aspects of regulation and repair. These effects position azithromycin to have a profound effect on inflammatory conditions in which the immune response contributes to detrimental tissue damage, organ failure, and death.

The emergence of severe acute respiratory syndrome (SARS)-coronavirus 2 (CoV-2) has thrust azithromycin into the spotlight due to early reports of improved outcomes in patients treated with azithromycin and hydroxychloroquine (17). The immunopathology of Coronavirus Disease 2019 (COVID-19) that results from SARS-CoV-2 infection is highlighted by weak innate antiviral responses as a result of inadequate production of the antiviral cytokines (type I and type III interferons), and robust pro-inflammatory responses with high levels of chemokine and cytokine expression (18). In some patients infected with SARS-CoV-2, pulmonary interstitial fibrosis results due to an overactive immune response to the infection (19). Furthermore, severe cases of COVID-19 are characterized by cytokine storm and acute respiratory distress syndrome (ARDS) requiring the need for immunosuppressive therapy and mechanical ventilation (20). The clinical evidence and immunopathology of SARS-CoV-2 indicate that infection drives an altered immunity in some individuals resulting in an overactive pro-inflammatory response, which invites the opportunity to treat severe cases with therapies capable of re-balancing the immune system.

**Figure 1:**
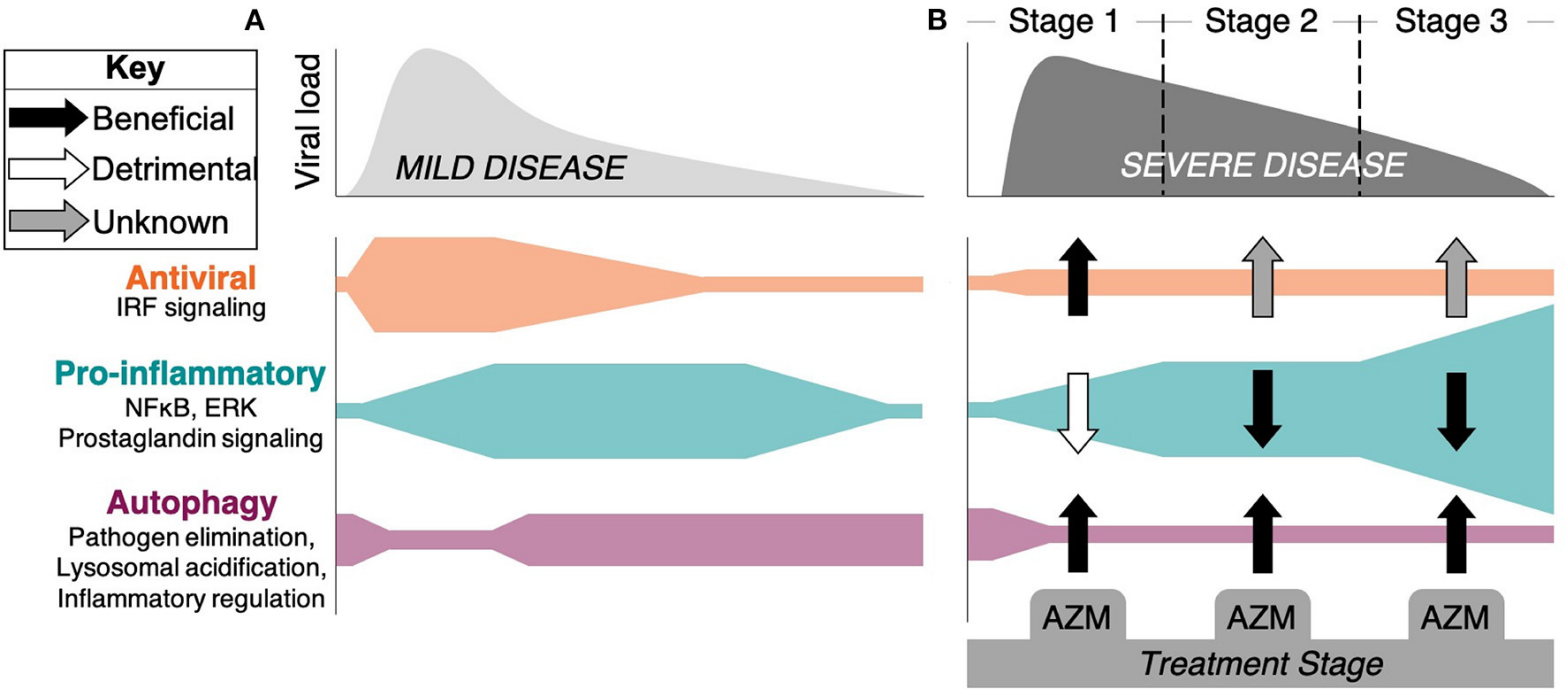
Stages of response and progression of SARS-CoV-2 infection and the potential impact of azithromycin therapy. A hypothetical timeline of viral burden kinetics and the associated immune response mechanisms are depicted for patients with **(A)** mild disease and **(B)** severe disease that is associated with organ damage, hypercoagulation, and death. **(A)** Antiviral responses coordinated through the detection of virus via pattern recognition receptors triggers IRF signaling and interferon production, along with pro-inflammatory signaling through NF-κB and ERK pathways. This initiates innate and adaptive immune mechanisms that limit viral spread and leads to mild symptoms and recovery. Autophagy plays a role in pathogen elimination, but can be inhibited by the virus. **(B)** In some patients, viral burden persists, possibly due to SARS-CoV-2 inhibition of IRF signaling pathways. Severe disease progresses through Stage 2 (lung damage) and Stage 3 consisting of hyperinflammation, cytokine storm, and hypercoagulation represented here by excessive NF-κB and other inflammatory pathway activity. Rampant inflammation that includes macrophage, neutrophil, and T lymphocyte driven pathology persists independent of viral control in Stage 3. The potential beneficial (black arrows), detrimental (white arrows), or unknown (gray arrows) impacts of azithromycin (AZM) are depicted as defined by evidence generated in other disease models suggesting that the drug could: increase type I/III interferon production; induce regulatory function of macrophages, blunt neutrophil influx, and decrease inflammatory cytokine production through inhibition of NF-κB signaling and other inflammatory pathways; and impact autophagy through blocking the degradation of autophagosomes that can impact virus infectivity, elimination, and the regulation of inflammation.

The clinical observations and data from COVID-19 patients support this premise. Many therapies are being investigated that suppress the overactive immune response (21), but the impact on immune mechanisms within these subjects is poorly defined. Azithromycin modulates the immune response through distinct pathways that may provide additional benefit by promoting repair rather than full immunosuppression. Here we review the immunomodulatory mechanisms of azithromycin along with its clinical use as an immunomodulatory therapeutic. We then discuss the potential impact of azithromycin on the immune response to COVID-19, highlighting mechanisms that potentially could provide therapeutic benefit, as well as cautioning of possible immunosuppressive activity and off-target effects including cardiotoxicity in these patients (Figure 1).

## COVID-19 and Current Immunotherapy

### Novel Coronaviruses

Coronaviruses (CoV) are enveloped, single stranded RNA viruses capable of infecting a range of hosts including humans, with the novel CoV's resulting in potentially fatal lower respiratory tract infection (22). The three most significant CoV outbreaks to impact humans include SARS-CoV in 2002, Middle East Respiratory Syndrome (MERS)-CoV in 2012, and most recently SARS-CoV-2 in 2019. The interplay between viral diversity, host species, and underlying clinical characteristics make CoV infections a challenge to predict, which is further compounded by the globalization and rapid escalation to pandemic levels. SARS-CoV-2 is an enveloped virus consisting of a lipid bilayer and four structural proteins, including spike (S), membrane (M), envelope (E), and nucleocapsid (N) proteins. The N protein is in complex with single-stranded RNA on the interior of the virus, while M and E are transmembrane proteins embedded in the lipid bilayer. S protein is anchored in the lipid bilayer and forms a protein corona that engages with target receptors for cellular entry (23). Notably, SARS-CoV and SARS-CoV-2 enter cells after binding angiotensin converting enzyme 2 (ACE2) while MERS enters through dipeptidyl peptidase 4 (DPP4) (24). Both receptors are expressed throughout the body and are upregulated in subjects with comorbidities, leading to increased severity of infection in some subjects (25, 26). These characteristics lead to additional complications that compound the health outcomes in high risk populations. At the time of the submission of this review, SARS-CoV-2 has infected over 90 million people across the globe and has contributed to over 1,950,000 deaths as the global pandemic continues to evolve.

### Stages of the Immune Response

COVID-19 has loosely been characterized to be comprised of 3 immunological stages. In the first stage, an interferon response coordinates the control of viral replication. The second stage is characterized by suppression of interferons by the virus, leading to lung damage and progressive disease. Some patients progress to a third stage of hyperinflammation coordinated by excessive macrophage activation and coagulation (27). In later stages, infected cells die and release virus particles along with intracellular components that stimulate an exaggerated innate response accompanied by large amounts of pro-inflammatory cytokine production. Adaptive responses are then triggered late, including CD4+ T cell cytokine release, CD8+ T cell mediated cytotoxicity, and B cell production of antibodies. The recruitment of inflammatory monocytes and macrophages to the lungs lead to a hyper-inflammatory state which contributes to the depletion of lymphocytes and induces a cytokine storm (28, 29).

The host response is therefore implicated as a pathologic factor of disease progression. Animal models of SARS infection demonstrate that lung inflammation worsens after viral clearance and peaks several days later (30). This suggests that the clinical deterioration in late stages of the disease is likely driven not by the virus, but by uncontrolled immune responses (31–33). Although there remains much to be learned, it seems helpful to define the clinical pathology of COVID-19 in separate stages, as the responses that are suppressed by the virus in the early stages of infection are the very same that are involved in the late hyperinflammatory state and are associated with increased severity and mortality. Therefore, caution should be taken when utilizing azithromycin or any other treatments that modulates immunity, due to the potential to suppress antiviral immune mechanisms. Whether immunomodulatory therapy in an individual patient is helpful or harmful likely depends on whether there still exists a high viral titer or whether the individual is in the later phases of hyperinflammation.

### Current Immunotherapies

Multiple clinical trials are currently being conducted that include agents that suppress immune function, with some being used clinically despite the lack safety or efficacy data. Many of the inflammatory mechanisms being targeted in these trials are associated with macrophage activation or macrophage effector functions (34). These targets include inflammatory cytokines such as IL-6, IFNγ, IL-1β, and TNFα; intracellular signaling pathways including JAK-STAT signaling, TLR signaling, and the inflammasome; and chemokines including CCR2 and CCR5 (34). Without a thorough understanding of the immunopathology of COVID-19, these therapies could have detrimental effects depending on which stage of disease and what level of immune hyperactivation a patient is currently experiencing. Poorly-timed immunomodulation could contribute to the failure of viral clearance, or to the inhibition of immunoregulatory mechanisms that balance the destructive components of the response.

Initially, azithromycin was used to prevent bacterial super-infections in early clinical studies evaluating the potential anti-viral effects of hydroxychloroquine in patients with COVID-19. Soon after SARS-CoV-2 reached pandemic status, 2 non-randomized studies were published, suggesting that hydroxychloroquine in combination with azithromycin reduced viral load in patients with COVID-19 (17, 35). As a result, the U.S. Food and Drug Administration authorized emergency use of hydroxychloroquine for the treatment of COVID-19, and it was being widely used alone or in combination with azithromycin despite limited evidence. Hydroxychloroquine is an anti-malarial agent that inhibits acidification of endosomes thus preventing viral replication *in vitro* (36). Indeed, the initial clinical reports showed reduced viral loads with hydroxychloroquine in patients infected with SARS-CoV-2, an effect which was further enhanced with azithromycin (17). However, subsequent clinical studies contradicted these findings demonstrating no added anti-viral effects with combination therapy, emphasizing the need for additional studies to define any potential synergistic effects or increased risks (37). In a retrospective cohort study conducted in 368 United States veterans, hydroxychloroquine with or without the addition of azithromycin showed no benefit in the treatment of patients hospitalized with COVID-19, and of concern, the group receiving hydroxychloroquine alone had a significantly higher mortality rate (38). An additional single-arm study of 11 subjects treated with hydroxychloroquine and azithromycin reported no impact on virologic clearance (37). Subsequently, a retrospective analysis of 1,061 patients with COVID-19 treated with hydroxychloroquine and azithromycin in France reported virologic cure in over 90% of the cohort within 10 days of the initiation of therapy (39). Results from these reports are difficult to interpret, as there is no way to factor in treatment bias or underlying severity of cases, which could significantly skew the reported results. At present, a high number of clinical trials are planned or underway evaluating chloroquine or hydroxychloroquine for the treatment of COVID-19, many of which also include these agents in combination with azithromycin. One such study recently published demonstrated a reduction in mortality in patients hospitalized with COVID-19 when treated with hydroxychloroquine alone or in combination with azithromycin (40). Due to disparate results, clearly additional trials are needed. The potential immune targets in response to SARS-CoV-2 infection, and the evidence suggesting a potential impact of azithromycin for each is summarized in Table 1.

**Table 1:** Host responses of COVID-19 and the potential immunomodulatory targets of azithromycin, along with a summary of the supporting evidence and species investigated (m, mouse; hu, human).

**COVID response**	**Potential target**	**Azithromycin effect and supporting evidence**			**Species**
Weakened type I/III IFN	Type I/III IFN	**↑**		Upregulates *in vitro* production in response to rhinovirus (41–43) and Zika virus (44) infections	hu
Cytokine Storm	IL-6		**↓**	Decreases *in vivo* concentrations in *P. aeruginosa* and *S. pneumoniae* pneumonia (45, 46) and after AMI (47)	m
			**↓**	Decreases serum concentrations in CF (2–6) and after influenza infection in combination with oseltamivir (48)	hu
	TNFα		**↓**	Decreases serum concentrations in CF (2–6)	hu
			**↓**	Decreases *in vivo* concentrations after SCI (49) and after AMI (47)	m
	IL-1		**↓**	Decreases *in vivo* concentrations after SCI (49) and after AMI (47)	m
	IL-8		**↓**	Decreases serum concentrations after influenza infection in combination with oseltamivir (48), in BOS (50, 51), and in transplant recipients experiencing acute allograft dysfunction (52)	hu
			**↓**	Blocks IL-17-induced *in vitro* IL-8 production in airway smooth muscle cells (53)	hu
Inflammatory monocytes/macrophages	NF-κB		**↓**	Inhibits nuclear translocation of NF-κB subunits (7–11)	m, hu
			**↓**	Associated with decreased inflammatory cytokine production (7–9, 11, 54–56)	m, hu
	STAT1		**↓**	Inhibits *in vitro* phosphorylation of STAT1, classical macrophage activation (12)	m
	Arg-1	**↑**		Increases expression which controls production of NO and T cell proliferation (45, 57, 58)	m
	Inflammasome		**↓**	Decreases *in vitro* mRNA stability for the NLRP3 gene transcript (13)	hu
Neutrophils	Recruitment/influx		**↓**	Decreases numbers in peripheral blood and inflamed tissues in CF (59) and BOS (50, 52)	hu
			**↓**	Decreases influx into inflamed tissues in bacterial pneumonia (45, 60), AMI (47), and BOS (61)	m
	NET formation		**↓**	Decreases NET release *in vitro* (62)	hu
	IL-1β		**↓**	Inhibits AP-1 signaling in lungs induced by LPS administration (63)	m
Lymphocytes	NF-κB		**↓**	Inhibits *in vitro* NF-κB pathway in T cells (erythromycin) (64)	m
	mTOR pathway		**↓**	Suppresses CD4+ T cell activation through modification of downstream targets in mTOR pathway (65), possibly in NK cells to decrease inflammatory cytokine production (66)	m, hu
	IL-17		**↓**	Decreases lung concentration in BOS-induced lung transplant (61)	m
			**↓**	Decreases plasma concentrations after influenza infection in combination with oseltamivir (48)	hu
Autophagy	Autophagosomes	**↑**		Increases number and alkalinizes lysosomes in macrophages (15, 16)	hu
	TNFα/IFNγ		**↓**	Inhibits expression, thereby reducing autophagosome clearance (15)	hu

The potential cardiotoxicity of azithromycin and other immune-based therapies should be considered and assessed in the ongoing clinical trials evaluating therapeutic efficacy against SARS-CoV-2. Patients with underlying comorbidities are at significantly higher risk of hospitalization and severe disease, with cardiovascular pathology and its complications among the leading causes of death in patients with COVID-19 (67). Acute cardiac injury is commonly observed in severe cases and is strongly associated with mortality in 19–40% of hospitalized COVID-19 patients (31, 67, 68). In addition to multi-organ failure and ARDS, patients that experience cytokine storm also have a high incidence of myocardial injury and cardiomyopathy (69, 70). Pro-inflammatory monocyte and macrophage recruitment along with an increased production of the chemokines MCP1 and IL-8 are associated with mortality among COVID-19 patients (71). These changes are reminiscent of the inflammatory response after acute myocardial infarction (AMI) which is characterized by an imbalance between pro- and anti-inflammatory macrophages leading to cardiac dysfunction and impaired healing. Although hydroxychloroquine and azithromycin in combination have shown potential efficacy in enhancing viral clearance, patients with COVID-19 have higher incidence of fatal arrhythmias and heart failure, either due to pre-existing conditions or to the infection itself. As discussed below, azithromycin may have utility in decreasing the inflammatory mediators associated with cytokine storm; however, because of the drug's association with cardiotoxicity and arrhythmia (72–76), particularly when combined with hydroxychloroquine (77), its use in COVID-19 patients should proceed with caution. Future modifications in the manner in which these agents are utilized may be required to maintain their therapeutic potential while reducing adverse consequences in the most vulnerable patients.

## Azithromycin

### Overview

Azithromycin is a macrolide antibiotic primarily used in the treatment of upper and lower respiratory tract infections that is effective against some Gram-positive, Gram-negative, and atypical bacteria through binding to the 50S ribosomal subunit in bacteria thereby inhibiting protein synthesis (78). Azithromycin is orally bioavailable and accumulates within cells and tissues, particularly in macrophages, to achieve tissue concentrations that are 50-fold greater than that in plasma (79, 80). Additionally, azithromycin has a long half-life estimated to be about 35–40 h in humans following a single 500 mg dose (78). Despite its large therapeutic window, azithromycin can cause gastrointestinal toxicity and cardiotoxicity, including QT prolongation and arrhythmia (81). Characterization of this off-target effect in clinical studies led the U.S. Food and Drug Administration to issue a warning concerning the cardiotoxicity of azithromycin (72–76). Despite this risk being very low in patients with no co-existing risk factors, the use of azithromycin should be closely monitored in patients with pre-existing cardiac problems, arrhythmias, baseline QT prolongation, and electrolyte disturbances (73–76, 82).

### Clinical Use of Azithromycin as an Immunomodulator

Azithromycin is also utilized clinically to modulate immune responses, primarily in patients with chronic inflammatory lung diseases. The same anti-infective dosage range of 250–500 mg was utilized in the vast majority of studies evaluating its immunomodulatory impact. However, several of the trials described below demonstrate that instead of daily dosing, thrice weekly administration is also effective, likely due to its accumulation within macrophages and long half-life (78–80). Azithromycin therapy has been demonstrated to improve lung function in patients with panbronchiolitis (83, 84), and decrease the rate of pulmonary functional decline in patients with bronchiolitis obliterans syndrome (BOS) after lung transplantation (50, 85–87). Azithromycin therapy also has been established in a series of randomized trials to decrease the frequency of pulmonary exacerbations and improve quality of life measures in patients with chronic obstructive pulmonary disease (COPD) (88, 89). This benefit appears to be most applicable to subsets of patients who are older and those with more mild disease (90, 91). Trials in patients with asthma have produced mixed results (92–95), and a recent meta-analysis of 7 randomized controlled trials demonstrated no beneficial clinical outcomes attributable to chronic azithromycin therapy (96). Effectiveness in these patient populations is thought to mainly be due to the ability of azithromycin and other macrolides to reduce pro-inflammatory cytokine production and decrease neutrophil influx, although the antimicrobial and antiviral effects also likely contribute.

The vast majority of azithromycin's evaluation and use as an immunomodulatory therapeutic, however, has been conducted in patients with cystic fibrosis (CF). Patients with CF suffer from chronic lung inflammation due to immune dysregulation and thickened mucus in the lungs caused by mutation of the cystic fibrosis transmembrane conductance regulator (CFTR) gene that impacts proper chloride ion transport (97). A series of randomized, placebo-controlled clinical trials established the short-term efficacy and safety of longitudinal azithromycin therapy in patients with CF (2–6). Additionally, a meta-analysis of these trials was conducted comparing azithromycin therapy against placebo by including 959 patients spanning a wide age range (98). Beneficial effects included significant improvement in pulmonary function, a reduced frequency of exacerbations, a decrease in hospitalizations, and an improved quality of life. Recently, the first long-term study of azithromycin therapy in patients with CF was published (99). This observational analysis showed that azithromycin reduced pulmonary functional decline over a 3 year period, with a significantly more pronounced effect in patients chronically infected with *Pseudomonas aeruginosa*, the organism responsible for the highest incidence of acute pulmonary exacerbation in these patients (99).

These clinical studies demonstrated that azithromycin blunts neutrophil influx into the lungs, an effect associated with decreases in IL-8, neutrophil elastase, and C-reactive protein concentrations (3, 59). Despite the immune modulation that could have led to a decreased ability to effectively respond to pathogen invasion, and although long-term antibiotic use could contribute to antimicrobial resistance and other issues of collateral damage, the chronic use of azithromycin reduced infection risk and the need for antibiotics (98). Patients with CF treated with azithromycin had significantly lower rates of *Staphylococcus aureus* and *P. aeruginosa* infections, and with the exception that use of the drug was associated with a higher number of infections with macrolide-resistant *S. aureus*, the risk for acquiring all other infections was not impacted (98).

### Immunomodulatory Mechanisms of Azithromycin

#### Antiviral Effects of Azithromycin

The impact that azithromycin exposure exerts upon viral replication and survival has been demonstrated for a number of viral pathogens (41, 100). Antiviral effects are most likely due not to the direct binding of azithromycin to viral targets, but alterations in mammalian cellular functions that disrupt the mechanisms by which viruses replicate, spread, and survive. One hypothesis of the drug's antiviral effect is through its ability to increase the pH in endosomes (101). Azithromycin is a weak base and accumulates in endosomal vesicles and lysosomes, which could increase the pH and block endocytosis and viral shedding (101). Additionally, azithromycin blocks internalization of influenza virus by host cells during early phases of infection *in vitro*—this was translated to a mouse model of influenza infection in which azithromycin reduced viral loads after a single intranasal administration (102). Similarly, there exists the potential for azithromycin to inhibit the entry of SARS-CoV-2 by interfering with the binding of the virus to its receptor, ACE2, but this has only been proposed using quantum mechanical energetics modeling (103). Additionally, the inhibition of autophagosome clearance by azithromycin in human cells discussed below could impact viral disposition inside infected cells (15).

Azithromycin can also exert antiviral effects through the up-regulation of interferon production. In a study using normal bronchial epithelial cells, azithromycin significantly increased rhinovirus 1B- and rhinovirus 16-induced interferons and interferon-stimulated gene mRNA expression and protein production (41). Replication and release of each of the rhinoviruses tested was significantly reduced at biologically achievable concentrations of azithromycin after 24 and 48 h of culture (41). Similar findings were reported using primary bronchial epithelial cells from children with CF and from adults with COPD (42, 43). Azithromycin augmented the expression of type I and III interferons, along with retinoic-inducible gene I (RIG-I)-like helicase, a viral pattern recognition receptor that leads to interferon signaling, in bronchial epithelial cells isolated from COPD patients infected with rhinovirus 16 (43). Rhinovirus infections are a primary cause of virally-induced respiratory exacerbations in patients with COPD and CF (104, 105), and therefore longitudinal azithromycin therapy may impact exacerbation frequency through this mechanism. Additionally, azithromycin was shown to attenuate the replication of Zika virus by the same mechanism associated with the up-regulation of the production of host type I and III interferons (44).

#### Azithromycin Inhibits Inflammatory Cell Signaling

The anti-inflammatory properties of the macrolide antibiotics were first investigated by exploring the impact of erythromycin on the suppression of cytokine production (106, 107). This led to studies examining the ability of macrolides, including azithromycin, to suppress the activation of the inflammatory transcription factor NF-κB. These early studies demonstrated that azithromycin prevents the nuclear translocation of the activated subunits of NF-κB thereby reducing the up-regulation of pro-inflammatory gene expression (7–11). These data led to the evaluation of the impact of azithromycin upon other aspects of inflammatory cell signaling including suppression of the inflammasome, inhibition of phospholipase-A2 (PLA2), and the regulation of autophagy (13–15, 108). Decreases in NF-κB DNA binding were mechanistically linked to the suppressed induction of pro-inflammatory genes and cytokine production in different murine and *in vitro* models of inflammatory and infectious diseases (7–9, 11, 54–56). The impact of azithromycin on cytokine and chemokine production impacts downstream inflammatory processes including a reduction in immune cell infiltration, alterations in epithelial cell barrier function, and decreases in endothelial cell expression of adhesion molecules (11).

Erythromycin was first demonstrated to inhibit NF-κB DNA binding in human Jurkat T cells (64), and to inhibit the DNA binding of both NF-κB and activator protein (AP)-1 in human bronchial epithelial cell lines stimulated by phorbol esters (109). These mechanisms were subsequently demonstrated in human monocytes stimulated with lipopolysaccharide (LPS) using the macrolide clarithromycin (110), and then with azithromycin in CF- and non-CF human bronchial epithelial cell lines (7, 111). Additionally, a study using cells isolated from the tracheal aspirates of neonates demonstrated that the decrease in NF-κB activation (and subsequent IL-6 and IL-8 production) by azithromycin is associated with inhibition of the degradation of IκBα, the protein that prohibits the active subunits of NF-κB from translocating into the nucleus (112). These effects are associated with a significant reduction in inflammatory cell infiltration into infected lungs, and a profound decrease in pro-inflammatory cytokine concentrations in the alveolar space.

Azithromycin also inhibits LPS-induced expression of PLA2. This enzyme is involved in cell signaling processes that produce arachidonic acid and eicosanoids (113), and also induces cytokine and chemokine production in macrophages, neutrophils, and endothelial cells (114). In a mouse macrophage cell line stimulated with LPS, azithromycin decreased eicosanoid and arachidonic acid production, along with IL-6 and prostaglandin E2 (108). From this work and additional results that demonstrate macrolides bind to membrane-bound phospholipids, the authors hypothesized that decreases in PLA2 substrate availability contributes to the anti-inflammatory mechanism of these agents (108, 115).

Additionally, several groups have studied the impact of azithromycin on ERK1/2 and inflammasome function. AP-1 activity is regulated in part by ERK1/2 signaling molecules (116), and binding of AP-1 to the enhancer region of the IL-1β gene is important for its expression (117). While NF-κB activation is necessary for the gene transcription of inactive forms of inflammatory cytokines such as pro-IL-1β, cleavage by the caspase-1 inflammasome is required for activation (118). Azithromycin was reported to inhibit AP-1 signaling in neutrophils isolated from the lungs of mice induced by LPS administration, which thereby decreased IL-1β concentrations (63). This effect on the inflammasome is also associated with the drug's ability to decrease the mRNA stability for the NLRP3 gene transcript, a sensing component of the NALP3 inflammasome, in human monocytes (13). Although the interplay between these signaling pathways is clearly impacted by azithromycin, the primary mechanism of action and target of azithromycin remain undiscovered.

#### Azithromycin Alters Macrophage Polarization

Studies of the immunomodulatory properties of azithromycin were extended to define the agent's impact on macrophage polarization. Macrophages are polarized to distinct functional phenotypes via signaling through two separate pathways. Classical, or M1 macrophages are activated by TNFα or IFNγ when stimulated by non-self foreign antigens, and through signaling via STAT1 and NF-κB pathways induce an inflammatory gene expression pattern (119, 120). Conversely, alternatively (M2)-polarized macrophages are activated by IL-4 or IL-13, are involved in Th2-type inflammatory responses, and function to orchestrate remodeling and repair and to regulate inflammation (121, 122). While the M1/M2 macrophage polarization paradigm oversimplifies the dynamic and complex macrophage activation landscape (123), it is a useful framework for comparisons across different disease states, treatments, and between *in vivo* and *in vitro* systems. Experiments using the murine macrophage cell line J774 demonstrate that azithromycin polarizes macrophages to an M2 alternative-like phenotype *in vitro* (57). In macrophages polarized to an M1 phenotype with IFNγ and LPS, azithromycin inhibited pro-inflammatory cytokine expression (including IL-12 and IL-6) and shifted surface receptor expression to that typically observed in alternatively-activated macrophages. Additionally, expression of the M1-effector protein iNOS was decreased and expression of the M2-effector protein arginase was increased by the drug (57). The effect of azithromycin on macrophage polarization has also been demonstrated in human monocytes stimulated to undergo classical activation *in vitro* (9). Azithromycin inhibited production of the M1 macrophage proteins CCR7 and IL-12p70, but in this study TNFα and IL-6 production were unaffected. The drug also increased M2 protein expression including IL-10 and CCL18 in this model (9).

This was followed up by studies investigating macrophage polarization by azithromycin in mice infected with *P. aeruginosa*. Regulatory macrophage functions were induced by azithromycin treatment early after infection with *P. aeruginosa*-laden agarose beads, a model that causes a prolonged, sub-chronic pneumonia (124). This led to a reduction in neutrophil influx, decreased inflammation, and reduced fibrotic lung damage that correlated with improved morbidity and survival (45). Macrophage polarization toward a more regulatory response was reflected in that M2-associated surface expression of mannose receptor (MR) was increased, as was the production of arginase (45). Additionally, infiltrating monocytes and macrophages in the azithromycin-treated mice exhibited greater production of IL-10 and significantly lower production of TNFα, CCL2, and IL-6. Importantly, the clearance of *P. aeruginosa*, an organism that is not susceptible to the drug, was not altered by azithromycin treatment. This work demonstrates that modulation of neutrophil and inflammatory macrophage-driven inflammation can be regulated by azithromycin without mitigating the ability of the immune response to evoke bacterial clearance (45). These results are reflective of the clinical practice of longitudinal azithromycin therapy in patients with CF, in whom *P. aeruginosa* often drives exacerbations and pulmonary functional decline (2–4).

A recent study shed additional light on the mechanism by which azithromycin polarizes macrophages to a regulatory phenotype (12). Incubation of a murine macrophage cell line or primary murine macrophages with azithromycin was found to increase the overall expression of IκB kinase (IKKβ), a molecule involved in signaling to NF-κB activation. When cells were stimulated with IFNγ and LPS, azithromycin treatment increased the phosphorylation of IKKβ despite a reduction in the subsequent signaling resulting in inhibition of NF-κB translocation into the nucleus (12). The kinase activity of IKKβ was inhibited by azithromycin and represents a potential mechanism for these effects. Because of a previous report demonstrating that the over-expression of IKKβ can inhibit STAT-1 signaling (the pathway responsible for classical macrophage activation in the presence of IFNγ) (46), investigators then explored this connection and found that azithromycin inhibited the phosphorylation of STAT-1, an effect that was dependent upon IKKβ. Induction of the M2 protein arginase was also dependent on this cross-talk, as IKKβ inhibitors reversed the ability of azithromycin to induce arginase activity (12). Likewise, macrophage-specific deletion of IKKβ conferred resistance to Group B Streptococcus infection in mice, an effect that was associated with increased expression of inflammatory molecules including IL-12, iNOS, and MHCII (46). This work provides a possible mechanistic link between NF-κB signaling inhibition and macrophage polarization by the drug.

Macrophage polarization with azithromycin has also recently been demonstrated to be effective in reducing inflammatory injury stemming from acute myocardial infarction (AMI) (47, 125) and spinal cord injury (126). Myocardial and spinal cord injury are exacerbated by the recruitment of monocytes, macrophages, and neutrophils in response to tissue damage (127, 128). In the case of AMI, neutrophil and inflammatory macrophage infiltration is associated with ineffective tissue remodeling and the development of heart failure (129, 130). In a mouse model of permanent coronary artery ligation, azithromycin treatment polarized the macrophage response to an M2-dominant phenotype and blunted the influx of neutrophils into the heart after AMI, thereby decreasing scar size, cardiac remodeling, and mortality (47, 125). Similarly, azithromycin treatment after SCI in mice was shown to increase markers of regulatory macrophage activity, improve locomotor recovery, and reduce SCI lesion pathology (49, 126, 131). In each of these conditions, mice treated with azithromycin had significant decreases in pro-inflammatory macrophage effectors including TNFα, IL-6, IL-1β, and MCP1, and an increase in M2-associated expression of CD206 and TGFβ. This work is supported by gene expression data generated using *in vitro* systems that broadly captures the immunomodulatory shift in macrophage polarization by azithromycin (49). Similar observations using azithromycin have also been reported in other neuroinflammatory conditions including stroke and retinal ischemia/reperfusion injury (132, 133).

Clinical studies also reflect the impact of azithromycin therapy on macrophage polarization in patients with inflammatory diseases. Azithromycin administration to patients with COPD led to an increase in expression of the M2-associated protein MR, along with reduced airway inflammatory cytokine concentrations and improved phagocytic function of alveolar macrophages (134). And in a study of patients with systemic lupus erythematosus, in addition to causing a reduction of pro-inflammatory cytokine production, azithromycin increased the gene expression of the M2 macrophage effectors arginase-1, Fizz-1, and IL-10 (135). While preclinical and clinical studies of the therapeutic utility of alternative macrophage polarization in conditions with inflammatory pathology show promise, additional investigation is necessary in order to determine which regulatory aspects of macrophage function are beneficial.

#### Azithromycin Directly and Indirectly Impacts Neutrophils

The mechanisms described above demonstrate that azithromycin reduces damaging aspects of neutrophil-driven responses through its impact on the coordination of inflammation and repair by macrophages. Additionally, azithromycin can directly affect neutrophil function. Neutrophils, primed by damage-associated molecular patterns and other signals, play important, though often destructive, roles in airway diseases including CF (136, 137), asthma (138), COPD (139), and ARDS (140). Azithromycin accumulates in neutrophils and has an extremely long half-life in these cells (51, 141). *In vitro* azithromycin exposure of human neutrophils modulates neutrophil extracellular trap (NET) release (62), and a recent report demonstrated that the macrolide erythromycin decreases airway NET formation in mice (142). Azithromycin has also been shown in human clinical studies to decrease IL-8 release and neutrophil airway infiltration, cause degranulation and degradation of extracellular myeloperoxidase, and reduce neutrophil oxidative burst (50, 51). Macrolides also decrease production of leukotriene B4 (LTB4) (143), a potent neutrophil chemoattractant that stimulates neutrophil IL-8 release (144).

#### Azithromycin's Impact on Lymphocytes

While the alteration of cytokine production of myeloid cells can impact adaptive immunity in many ways, less is known about the direct effects of azithromycin and the other macrolides on lymphocyte function. Early studies of the immunomodulatory properties of erythromycin demonstrated that NF-κB inhibition also occurs in T cells when stimulated *in vitro* (64). Azithromycin has also been shown to suppress T cell activation through modification of the mTOR signaling pathway (65). A direct effect of azithromycin on T cells was characterized in a study of 11 subjects with COPD. Azithromycin therapy reduced granzyme B production in the airways, an effect that was confirmed *in vitro* to be suppressed in both CD4+ and CD8+ T cells (145). Macrolides also impact the Th1/Th2 cytokine balance, although reports are conflicting as to whether the shift is more toward the production of IFNγ or IL-4. In a study of patients with CF, clarithromycin therapy skewed the response toward that of Th1 dominance (146), and a study using *ex vivo* stimulation of primary mononuclear cells exposed to clarithromycin demonstrated an increase in the number of T cells producing IFNγ (147). However, in a similar study examining mitogen-stimulated human T cells *in vitro*, clarithromycin inhibited IFNγ production to a greater extent than it inhibited the production of IL-4 (66).

Little has been investigated in terms of a direct effect of azithromycin on natural killer (NK) cells. NK cells are innate effector lymphocytes that are important in the response to viral infections. *In vitro* studies demonstrate that azithromycin decreases perforin production in primary human NK cells as well as the production of IFNγ and TNFα in an NK cell line (148). Although the impact of azithromycin on NK cell disposition during infection remains to be studied, because the mTOR pathway governs NK cell activation as it does in T cells, azithromycin may have a significant impact on the ability of NK cells to produce cytokines or other functions that contribute to either the inflammatory response or the clearance of pathogens.

Azithromycin also can dampen the lymphocytic airway inflammation associated with BOS after lung transplantation. IL-17 is important in the pathophysiology of allograft rejection, during which production of the cytokine by CD8+ T cells drives a lymphocytic airway inflammation (149, 150). Compounding this problem is the fact that regulatory T cells, which typically inhibit IL-17 production, are often reduced due to the immunosuppressive agents used to prevent rejection in patients who have undergone solid organ transplantation (151). In a study of 15 lung transplant recipients experiencing acute allograft dysfunction, azithromycin therapy decreased neutrophil and eosinophil influx into the lungs, blunted cytokine and chemokine production including IL-1β, IL-8, and CXCL10, and significantly controlled airway inflammation (52). While sputum concentrations of IL-17 were not affected, the authors concluded that azithromycin decreased IL-17+ T cell-mediated airway inflammation (52). Similar results were obtained in a separate study of 17 lung transplant patients with BOS, where azithromycin decreased neutrophil influx which was associated with decreased IL-8 mRNA expression, but no change in IL-17 mRNA expression was noted (50). This has additionally been recapitulated in mice subjected to IL-17 administration directly into the lungs (152), and in primary human airway smooth muscle cells in culture, in which azithromycin blocked IL-17-induced IL-8 production (53). Conversely, in an animal model of BOS induced by lung transplantation, azithromycin did significantly reduce the lung concentration of IL-17 as well as the number of IL-17-producing T cells (61). Additionally, a randomized clinical study evaluating the impact of azithromycin treatment in combination with oseltamivir for the treatment of influenza demonstrated that the addition of azithromycin decreased plasma concentrations of IL-17, IL-6, IL-8, CXCL9, and CRP (48). This was associated with faster symptomatic resolution, yet there was no change in viral clearance rates (48). Taken together, these studies suggest that azithromycin primarily affects T cell-mediated responses through an impact on downstream effectors, however a direct impact on IL-17 production is also probable.

When azithromycin polarizes macrophages to an alternative-like phenotype, this includes a dramatic increase in the production of the M2 protein arginase-1 (45, 57). Arginase-1 expression is induced in macrophages, neutrophils, and other immune cells where it competes with iNOS thereby regulating NO generation and limiting NO-mediated inflammatory injury (153). Additionally, arginase-1 can also immunoregulate by controlling T cell activation and proliferation (154, 155). T cells are highly sensitive to the localized depletion of arginine and other amino acids essential for their proliferation and function (154, 156, 157). Arginase-mediated depletion of arginine has been characterized to impact T cell-mediated responses in a variety of infection models and inflammatory processes including trauma, asthma, glomerulonephritis, and malignancy (158). In the case of viral infections, both arginase and NO can be either helpful or harmful through enhancement or inhibition of viral clearance, or through enhancement of the associated immunopathology (159). Control of T cell disposition through increased arginase activity represents an additional mechanism by which azithromycin could impact adaptive immunity, however more work in this area is needed.

#### Azithromycin Inhibits Autophagosome Flux

Autophagy, the catabolic process that regulates the elimination of defective intracellular proteins and components (160), also plays a complex role in both pathogen elimination and the regulation of inflammation (161). Azithromycin, at therapeutic concentrations, was demonstrated to increase the number of autophagosomes in macrophages (15, 16). Molecular studies in primary human macrophages determined that this occurs through the inhibition of autophagosome degradation, rather than by causing an increase in their synthesis (15). Azithromycin accomplishes this by inhibiting lysosomal acidification which thereby inhibits autophagosome clearance (15). Compelling evidence reveals that the dysregulated inflammatory state of myeloid cells with CFTR mutation is tied to defects in autophagy. Restoring efficient autophagy in mice with the F508del mutation in CFTR, the most common mutation that produces severe disease in CF, restores control of excessive production of pro-inflammatory cytokines by macrophages typically observed as a result of CFTR dysfunction (162–164). This discovery led to 2 clinical trials in patients with CF with F508del-CFTR mutations in which the restoration of autophagy resulted in the suppression of pro-inflammatory cytokine production and improved lung function (165, 166). However, inhibition of autophagy flux has also been linked to decreases in pathogen elimination (15, 167). While considerable overlap between autophagy and inflammation has been characterized, as autophagy contributes to both host defense and control of associated inflammatory injury (167, 168), the impact of azithromycin at this nexus remains to be studied.

## Immune Pathology of COVID-19 and Potential Impact of Azithromycin

### Antiviral Immune Response (Stage 1)

Upon exposure to CoV, the innate immune response is activated through standard anti-viral signaling cascades initiated through toll-like receptors (TLR), NOD-like receptors (NLR), and others, which result in signaling through pathways to activate downstream transcription factors including IRF3 and NF-κB (169). Typically, innate immune cells detect viral infection through these viral pattern recognition receptors which triggers the expression of type I interferon and other pro-inflammatory cytokines including IL-1, TNFα, and IL-6 (170, 171). The expression of type I interferon through IRF3 signaling is critical for the clearance of viral infections (172). The resulting influx of phagocytes to the site of infection initiates antigen processing and presentation which elicits both CD4+ (helper T cells) and CD8+ (cytotoxic T cells) responses in subjects infected with SARS-CoV or SARS-CoV-2. Coronaviruses are characterized by a phospholipid bilayer decorated with envelope proteins including spike protein (S), an internal nucleoprotein (N) in complex with RNA, and others (173). Durable and persistent antibody responses to the S protein have been reported in SARS-CoV infection (174) and MERS-CoV infection (175), with similar results observed thus far for SARS-CoV-2 (176–178). The receptor binding domain of the S1 spike protein is responsible for binding to ACE2, and is the likely target of eventual neutralizing antibody responses (179–181). These data indicate that both cellular and humoral immune responses are initiated to fight the virus.

Some patients infected with novel coronaviruses, however, fail to mount a robust initial response. This is thought to be due to evasive mechanisms of SARS-CoV, MERS, and SARS-CoV-2 that suppress early type I interferon production. SARS-CoV suppresses the activation of TNF receptor-associated factors (TRAF) 3 and 6, which suppresses early type I interferons and pro-inflammatory effectors including IL-6 and TNFα (182). The virus can also inhibit STAT transcription factor phosphorylation and thereby counteract type I IFN signaling (171). Notably, the N protein of SARS-CoV serves as an interferon antagonist and downregulates JAK-STAT signaling in infected cells (183). These mechanisms allow the novel coronaviruses to suppress immune responses thereby allowing proliferation of infection in larger numbers of pulmonary epithelial cells.

Additionally, activated CD8+ cytotoxic T lymphocytes and NK cells are important for viral clearance, and both of these cell types have been found to be significantly decreased in patients with more severe COVID-19 (184, 185). Although the mechanism is unknown, this is likely due to virus-induced apoptosis and decreased T cell priming and activation secondary to impaired dendritic cell migration found with SARS-CoV infection (68, 186, 187). These factors likely contribute to the propagation of the virus in patients that advance to severe disease.

The established ability of azithromycin to up-regulate the production of type I interferons in response to rhinovirus and Zika virus provides a foundation for investigating this mechanism in SARS-CoV-2 infection (41–44). Interferon λ1 (a type III interferon) is theorized to play a significant role in response to COVID-19 infection in humans by reducing viral load and preventing organ damage by preventing cytokine storm and the resulting tissue injury (188, 189). If azithromycin can induce the expression of interferon λ1, the drug could help augment interferon response to COVID-19 infection, improving viral clearance and reducing the potential for certain patients to progress to severe disease. However, caution should be used due to the possibility of suppressing other aspects of innate and adaptive immunity.

### Pathologic Inflammation (Stages 2 and 3)

#### Overview

**Figure 2:**
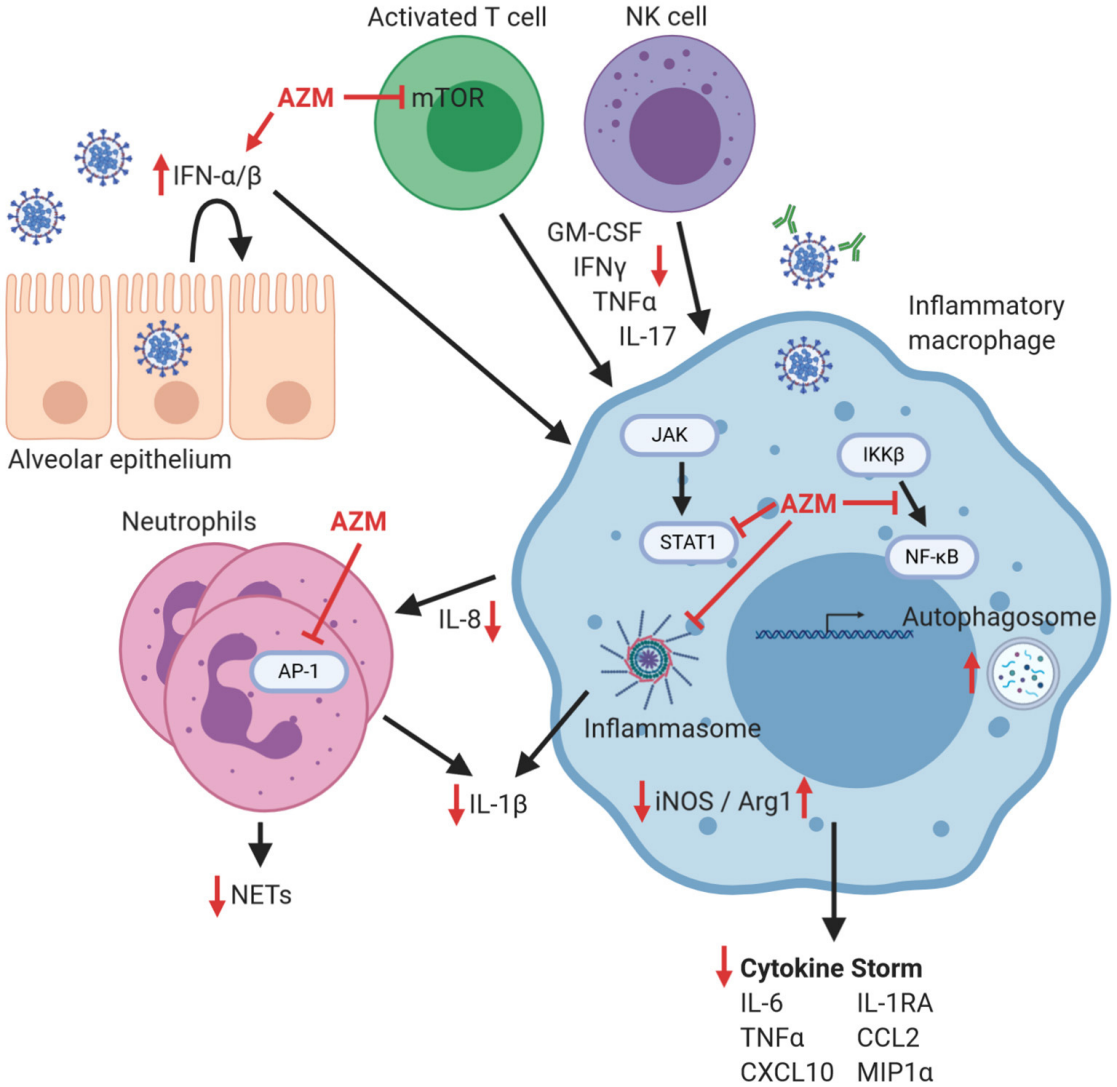
Potential effects of azithromycin on immune cells that contribute to hyperinflammation in COVID-19. The response to pulmonary infection with SARS-CoV-2 is characterized by a blunted Type I/II IFN response, which could possibly be improved by azithromycin (AZM). Macrophages are implicated in the coordination of the exaggerated inflammatory response that can lead to lung damage, cytokine storm, and increased morbidity. Azithromycin has been demonstrated in other models to inhibit signaling mechanisms in inflammatory macrophages including NF-κB nuclear translocation, STAT1 phosphorylation, and inflammasome activation that all contribute to pro-inflammatory mediator production including iNOS, cytokines, and chemokines. Neutrophil influx is inhibited by azithromycin, likely through impacting chemokine production and through direct inhibition of AP-1 signaling, leading to decreases in NET formation and production of IL-1β. Proliferation of activated T lymphocytes can be blunted by azithromycin through inhibition of mTOR signaling, as well as through increased macrophage production of arginase-1 (which thereby depletes arginine which is required for T cell proliferation). Consequently, T cell and NK cell production of inflammatory cytokines including GM-CSF, IFNγ, TNFα, and IL-17 would be decreased. Therefore, within this theoretical model lies the potential for azithromycin to enhance antiviral effects, blunt harmful hyperinflammation that leads to cytokine storm, or conversely inhibit desirable immunologic effects, depending on the phase of the antiviral response. Red inhibitory lines depict possible targets of azithromycin during COVID-19, and red arrows indicate resultant increases or decreases in the production of mediators of inflammation. Figure was created using https://biorender.com/.

The pathologic drivers of disease in patients infected with SARS-CoV-2 are not completely understood, but it is clear that severe disease is not only related to viral load. The dysregulated, excessive inflammatory response is thought to be a major driver of disease severity and death in patients with COVID-19 (27–29, 190). Patients with severe disease have high peripheral blood concentrations of pro-inflammatory cytokines, marked lymphopenia, and infiltration of inflammatory monocytes and macrophages in tissues throughout the body including the lungs, secondary lymphoid tissues, and the heart (191, 192). Increased numbers of neutrophils, decreased numbers of lymphocytes, and increased concentrations of serum inflammatory proteins including cytokines and chemokines have all been associated with worsened outcomes and death (71, 185, 193, 194). A recent report thoroughly characterized the gene expression associated with SARS-CoV-2 in human lung-derived epithelial cell lines, primary cultured human bronchial epithelial cells, infected ferrets, and COVID-19 patients (18). Compared to SARS-CoV and other viral infections, SARS-CoV-2 induces a unique response characterized by a paucity of type I and III interferon production and dramatic inflammatory cytokine and chemokine secretion that is independent of viral burden. By day 7 post-infection in ferrets, despite low virus levels, the transcription of cytokines and chemokines continues to expand (18). To support this, the addition of type I interferon to the culture of a bronchial epithelial cell line induced a dramatic reduction in viral replication, yet antagonism of type I interferon signaling by ruxolitinib had little effect on the production of inflammatory mediators (18). This suggests that the production of cytokines and chemokines induced by SARS-CoV-2 is independent of interferon-governed responses. Although much remains to be learned, the role of innate and adaptive immune cells in the resultant inflammatory pathology have been partially characterized. The contribution of the major immune cell populations, and the evidence of azithromycin's potential impact on each, are described below and depicted in Figure 2.

#### Macrophages

Pathologic inflammation in patients infected with novel coronaviruses is driven by high numbers of neutrophils, monocytes, and macrophages in the airways (185). Common features of the previous novel coronavirus outbreaks included dramatic inflammatory cell infiltration into the lungs leading to acute pulmonary injury and ARDS (195). Data from SARS-CoV and MERS outbreaks that show increased numbers of neutrophils and inflammatory monocytes in the airways of severely ill patients (196, 197). Likewise, previous studies also show that SARS-CoV infected patients that require intensive care have high plasma concentrations of CXCL10, CCL2, MIP1α, and TNFα, all of which are commonly produced by inflammatory myeloid cells (198). Similarly, most of the cells infiltrating the lungs in patients with severe COVID-19 are activated inflammatory monocyte and macrophages which produce pro-inflammatory cytokines and chemokines and activate coagulation (29, 199–202). Although macrophages serve as a front-line defense against invasive pathogens through the initiation and coordination of immune responses, they also regulate aspects of inflammation that are damaging to host tissues and promote remodeling and repair. Single-cell RNA sequencing of lung bronchoalveolar immune cells in patients with SARS-CoV-2 infection indicate that patients with severe disease, despite an increased myeloid cell percentage in the airways, have a depletion of resident alveolar macrophages, which typically serve a regulatory function, and an increased proportion of inflammatory monocyte-derived macrophages (203). Azithromycin's ability to promote regulatory macrophage characteristics could potentially restore the balance of inflammatory and regulatory macrophage phenotypes that are misaligned in patients with severe COVID-19.

The role of regulatory macrophage functions in response to SARS-CoV-2 is not well-understood, although data is beginning to emerge. A subset of macrophages from COVID-19 patients has been described as expressing a gene signature associated with tissue repair (203). Whether these functions are helpful, or whether they contribute to the promotion of fibrotic lung injury is not yet known. However, in a study of non-human primates, macaques acutely infected with SARS-CoV-2 demonstrated macrophage activation that included both pro-inflammatory and repair characteristics (204). The presence of anti-spike IgG prior to viral clearance decreased the regulatory aspects of macrophage polarization and promoted MCP1 and IL-8 production along with exaggerated monocyte recruitment to the lungs. This led to heightened lung injury and worsened outcomes, suggesting that the regulatory function of macrophages may be important in suppressing fulminant inflammation (204). If this is the case, the ability of azithromycin to promote the regulatory functions of macrophages may be beneficial in COVID-19 patients experiencing the detrimental aspects of myeloid cell-driven hyper-inflammation. In addition to inhibiting the production of inflammatory cytokines and chemokines, azithromycin promotes regulatory aspects of macrophage function including the production of IL-10, TGF-β, and arginase. As discussed above however, the work characterizing macrophage polarization by azithromycin has not been explored in the setting of a viral infection. Because the immunopathology is driven by inflammatory signaling pathways that include NF-κB, azithromycin's impact on macrophage polarization in COVID-19 is a reasonable hypothesis to explore.

However, animal studies of SARS-CoV demonstrate that M2 polarization and increased arginase-1 activity could be detrimental. In a mouse model of SARS-CoV infection, investigators demonstrated that alternatively activated macrophages were responsible for enhancing the pulmonary pathology (58). Previous studies by this group demonstrated that STAT1, a key signaling protein responsible for inflammatory macrophage responses, was necessary to control viral spread when infected with human SARS-CoV (205). SARS-CoV infection of mice lacking hematopoietic STAT-1 expression were shown to have greater morbidity and lung pathology, which was associated with the activation of M2 macrophages (58). To test whether the M2 macrophages were responsible for the enhanced pathogenesis, the authors generated STAT-1/STAT-6 double knockout mice, as STAT-6 drives M2 macrophage activation. With these mice, which did not mount an M2 macrophage response after infection, the extent of pulmonary pathology was normalized (58). Additionally, a separate group demonstrated that SARS-CoV infection in mice induces an immunosuppressive alveolar macrophage population that inhibits antiviral T cell responses (206). Therefore, M2 macrophage polarization with azithromycin, which may decrease inflammatory cytokine production and arginase-1 expression thereby regulating other damaging aspects of inflammation, could also be detrimental in patients infected with novel coronaviruses. Future investigation of the complex interplay between these cell types will be necessary in order to determine which therapeutic targets, and in what circumstances, treatment with azithromycin could be beneficial.

The autophagy-lysosomal system plays a central role during infection with SARS-CoV (207, 208). However, it is unknown whether the induction of autophagy may be beneficial to patients infected with SARS-CoV-2 (209). Autophagy is involved in viral entry, viral clearance, and both initiation and regulation of inflammatory pathways (167). There is conflicting evidence as to whether CoVs inhibit autophagy. Therefore, the inhibition of autophagosome flux by azithromycin could be beneficial in terms of direct antiviral effects, and could counteract the hyperinflammation associated with dysregulated pro-inflammatory cytokine release (15). Chloroquine, an immunotherapeutic agent being studied for its efficacy against SARS-CoV-2, also inhibits autophagic flux by inhibiting autophagosome-lysosome fusion (210). However, its mechanism of action in the case of SARS-CoV may not be due to this effect, but rather due to chloroquine's inhibition of endosomal acidification, thereby preventing cellular entry (36). Although the induction of autophagy, or the inhibition of autophagosome flux, could impact SARS-CoV-2 infection through multiple effects, a better understanding of the interaction between host mechanisms and the virus are needed in order to properly evaluate these targets.

#### Neutrophils

The ability of azithromycin to blunt macrophage-driven neutrophil influx lends promise to the drug's potential impact on patients infected with SARS-CoV-2. Recent reports concerning the immune response in COVID-19 have characterized extensive neutrophil infiltration into diseased lung tissue as well as significant evidence of NET release in the serum (211, 212). Much like NETosis observed in lungs of ARDS patients subsequent to pneumonia or sepsis, NET release in the lungs of COVID-19 patients may play a pathologic role (213). NETs have also been reported to promote intravascular coagulation (214), and although whether this impacts mechanisms that contribute to hypercoagulation and stroke that have been reported as clinical complications associated with COVID-19 remains to be determined (215, 216). Despite the usefulness of azithromycin in these neutrophilic airway diseases, and its direct inhibition of NET production, care should be taken when azithromycin is administered under the suspicion of viral infection. Rodent studies have found that neutrophils are protective during infection with SARS-CoV (217) and severe influenza (218, 219), and also help to prevent bacterial pneumonia secondary to influenza infection (220). Therefore, severely limiting neutrophil infiltration and activity in the airways may have some undesirable consequences, and the efficacy of such a therapy will likely depend on the individual patient circumstances.

#### Lymphocytes

Despite their depletion in COVID-19, lymphocytes appear to contribute to the macrophage hyperactivation that leads to the development of cytokine storm, a state in which pro-inflammatory cytokines drive excessive, damaging inflammation. Systemic cytokine profiles in patients with COVID-19 are similar to those observed in cytokine release syndromes often driven by macrophages, including high levels of circulating IL-6, IL-17, TNFα, CCL2, and CXCL10. These cytokines will induce T cells and NK cells to produce additional cytokines including GM-CSF and IFNγ. An abundance of peripheral blood CD14+CD16+ monocytes has also been described in COVID-19 patients with severe disease to produce high amounts of IL-6 (199, 221). In patients with severe COVID-19, this abundance was also associated with the presence of CD8+ T cells that produced high amounts of GM-CSF, which can further induce IL-6 production (221). Likewise, pathogenic CD4+ T cells producing both IFNγ and GM-CSF were exclusively found in patients with severe disease, indicating their likely role in the hyperinflammatory response (221). Additionally, results from a recent in-depth analysis of NK cells isolated from patients with COVID-19 revealed that despite low NK cell numbers in these patients, the NK cell phenotype associated with severe disease was robustly activated and associated with increased IL-6 levels (222). However, in a separate report, the presence of IL-6-producing macrophages was associated with severe lymphocyte depletion in the spleen and lymph nodes in patients with severe COVID-19 (223). Additionally, highlighting the complexity of these interactions, expression of genes and surface proteins associated with T cell and NK cell exhaustion has also been associated with severe disease (184, 221). As discussed above, modulating the immune response with azithromycin consistently results in decreased production of IL-6 across both infection- and non-infection-driven pathology. The drug's impact on IL-6 production could be a key factor in its potential efficacy, although the direct impact on NK cell production of IL-6 by azithromycin has not been studied.

The severity of disease for MERS-CoV, SARS-CoV, and SARS-CoV-2 has also been shown to positively correlate with levels of IL-17 and other Th17 cell-related pro-inflammatory cytokines (186, 224). A recent study of 39 patients with COVID-19 demonstrated that although CD4+ and CD8+ T cell numbers in circulation were low, the cells produced higher amounts of IL-17 when stimulated *in vitro*, which links lymphopenia to hyperinflammation (225). As discussed above, azithromycin may target T cells directly by inhibiting intracellular signaling pathways and expression of T cell cytokines including IL-17, although most of the effects on these immune mechanisms seem to center on the downstream effectors. Because IL-17 production by T cells and other cellular sources is associated with disease severity, targeting IL-17, which functions upstream of inflammatory cytokines that result in neutrophil recruitment, could be desirable, as these play major roles in the development of ARDS. Additionally, a Th17 dominant immune response has been reported to drive more severe viral myocarditis (226). If azithromycin does blunt IL-17 responses, it could impact morbidity and mortality related to COVID-19 virally-induced myocarditis. The studies that characterize the impact of azithromycin on IL-17-mediated pathology in lymphocyte-driven airway inflammation in BOS and influenza infection suggest promise associated with this mechanism (48, 50, 52, 61).

## Discussion

Based on the antiviral and immunomodulatory mechanisms presented, and based on the limited clinical evidence of its impact on viral clearance, the thorough evaluation of azithromycin as a possible treatment for patients with COVID-19 is warranted. The ability of azithromycin to impact the production of inflammatory mediators through its inhibition of NF-κB and other pro-inflammatory signaling pathways suggests the potential for benefit in counteracting the hyperinflammatory state that manifests through neutrophil influx, lung inflammation, cytokine storm, and hypercoagulation. It is likely that these immunomodulatory effects will be beneficial in patients infected with COVID-19, but careful evaluation of when to utilize the drug based upon current viral burden and immune status is critical. This approach has also been proposed in a recent communication published in The Lancet in which the authors recommend that patients with COVID-19 should be screened for hyperinflammation in order to identify the subgroup that may benefit from immunomodulatory or immunosuppressive therapies (190).

In conclusion, the immunomodulatory effects of azithromycin are complex and multifactorial. Impacting macrophage polarization, autophagy, and cytokine release will likely be beneficial in a subgroup of patients, but any treatment that impacts immune function should be used with caution in patients with an active infection. Until regulatory aspects of macrophage function are better defined in the setting of COVID-19, and until azithromycin and other therapies are properly evaluated in randomized clinical trials in defined patient populations, extreme caution should be exercised when utilizing azithromycin in these patients.

## Author Contributions

All authors listed have made a substantial, direct and intellectual contribution to the work, and approved it for publication.

## Conflict of Interest

VV, AA-L, JG, and DF have a patent pending for an azithromycin formulation to modulate immune responses. The remaining authors declare that the research was conducted in the absence of any commercial or financial relationships that could be construed as a potential conflict of interest.
